# Using “Diraya” System as a Complementary Tool in Nursing Process Education: A Controlled Clinical Study

**DOI:** 10.3390/jcm11102771

**Published:** 2022-05-14

**Authors:** Lourdes Díaz-Rodríguez, Keyla Vargas-Román, María del Mar Díaz-Rodríguez, Juan Carlos Sánchez-García, Antonio Liñán-González, Raquel Rodríguez-Blanque

**Affiliations:** 1 Department of Nursing, Faculty of Health Sciences, University of Granada, 18071 Granada, Spain; jsangar@ugr.es (J.C.S.-G.); rarobladoc@ugr.es (R.R.-B.); 2 FPU16/01437, Methodology of Behavioral Sciences Department, School of Psychology, University of Granada, 18071 Granada, Spain; keyvarom@ugr.es; 3 C.E.I.P. Cristo Rey, Consejería de Educación y Deporte, Junta de Andalusia, Jimena de la Frontera, 11320 Cádiz, Spain; maria.diaz.rodriguez.edu@juntadeandalucia.es; 4 Department of Nursing, Faculty of Health Sciences, Melilla Campus, University of Granada, 52005 Melilla, Spain; antoniolg@ugr.es

**Keywords:** academic health record system, case-based learning, competencies, Diraya, nursing process

## Abstract

Background: Healthcare has been revolutionized by the application of information and communication technologies. The implementation of electronic health record systems improves the quality and safety of patient healthcare. Nursing students who start learning the nursing process without contact with real patients experience difficulties in its correct application. Purpose: To compare the acquisition of skills and competencies in the nursing process by undergraduate nursing students between conventional learning with books and learning with an academic electronic health record system (Diraya). Methods: A controlled experimental study was conducted and included 379 students with a mean age of 20.54 ± 5.09 years, enrolled in the “Nursing Process and Basic Care” degree course at the School of Health Sciences in Granada. All participants gave their informed consent and were allocated by convenience sampling to a control group (*n* = 187; 21.20 ± 5.77 years) or an experimental group (*n* = 192, 19.91 ± 4.24 years). Findings: The experimental and control groups did not differ in sex distribution (*p* = 0.20), mean age (*p* = 0.01), or previous knowledge of the nursing process (*p* = 0.96). The groups did not significantly differ in multi-choice test results on the acquisition of theoretical knowledge (*p* = 0.13). However, the experimental group scored higher on clinical case planning (9.47 ± 0.80 vs. 8.95 ± 1.17; *p* < 0.001), took less time to complete it (46.9 ± 8.76 min vs. 82.66 ± 13.14 min; *p* < 0.001), and needed fewer autonomous learning hours to prepare for the final examination (2.26 ± 2.41 vs. 9.58 ± 3.83; *p* < 0.001). Satisfaction with the program and the rating of its quality were generally higher in the experimental group, while greater difficulty with most phases of the nursing process was reported by the control group. Conclusions: The academic electronic health record system “Diraya” is a useful tool to improve the learning and implementation of the nursing process by undergraduate nursing students.

## 1. Introduction

Information and communication technologies (ICTs) have revolutionized healthcare through the implementation of electronic health record (EHR) systems, which have improved the quality and safety of patient care [1]. In addition, there is evidence regarding nursing care frameworks that explains the positive influence of the use of ICT by nurses in their practice, showing that the interaction between nursing resources and nursing services can produce changes in patient conditions [2].

Over the past decade, all Spanish regional health services have made a major effort to develop more accessible and personalized systems [3]. In 2001, the public healthcare service of Andalusia (Southern Spain) implemented an EHR system, designated “Diraya”, to manage electronic clinical records and healthcare services (Diraya manual available at: https://www.huvn.es/profesionales/enfermeria/plan_de_acogida/enfermera/hospital_general/manejo_de_diraya, accessed on 15 January 2021). This system is used at all care levels in the Andalusian Health System as an integrated electronic clinical record system for all eight provinces in the region. It contains detailed information on the health of all patients attending the health centers and on the healthcare that they have received to date. These data are permanently available to healthcare professionals and are also used in the management of the regional health system. It currently oversees more than 8,000,000 clinical records (95% of the population) and provides support to more than 100,000 healthcare professionals [4].

Incorporation of EHR system knowledge and experience into nursing programs would increase the confidence of students in the management and utilization of these systems in the clinical setting [5,6]. Some educational institutions, such as the Universities of Central Lancashire and Wisconsin-Eau Claire, have developed Academic Electronic Health Record (AEHR) systems to enable students to practice the management of this technology, handling the documentation and simulating patient care planning [7,8]. One of the modules in the EHR Diraya system is specific to nursing, and the AEHR system simulates the external structure and functioning of this module; however, it does not contain clinical records, only generating randomized data on fictional patient names and hospital units.

Within the European Higher Education Area, greater focus on the quality of learning has led to the promotion of active teaching methods that encourage learner autonomy [9]. In this regard, case-based learning (CBL) has been developed in nursing education to maximize critical thinking abilities and decision-making skills, essential for high-quality care [10,11]. CBL uses inquiry-based learning methods in which students are confronted by real clinical cases to test their knowledge and prepare them for clinical practice [12].

The “nursing process” (NP) is a method used by nurses to deliver effective, efficient, personalized, and continuing care and is divided into five stages: assessing, diagnosing, planning, implementing, and evaluating. Learning the correct application of this approach is difficult in the absence of real patients, and nursing students need to be actively involved in resolving problems [13]. NP courses based on CBL could benefit from the use of an AEHR system to generate authentic cases and to prepare students for the management of this type of system [14,15]. The present study is highly relevant to nursing practice in the real-life clinical setting, where there is an increased utilization of AEHR systems. Nursing faculty and students should be familiar with their operation (e.g., data input procedures), and this study shows how an AEHR system could be used to improve the efficiency of learning in other nursing courses [16].

The objective of this study was to compare the acquisition of competencies by nursing students between a conventional teaching method using books and a novel approach using an AEHR system (Diraya).

## 2. Materials and Methods

### 2.1. Design

A prospective, controlled, experimental, open-label study was conducted using a convenience sample. For the results analysis, groups were labeled with a non-identifying term to minimize researcher expectation bias.

### 2.2. Setting and Selection of Participants

**Figure 1:**
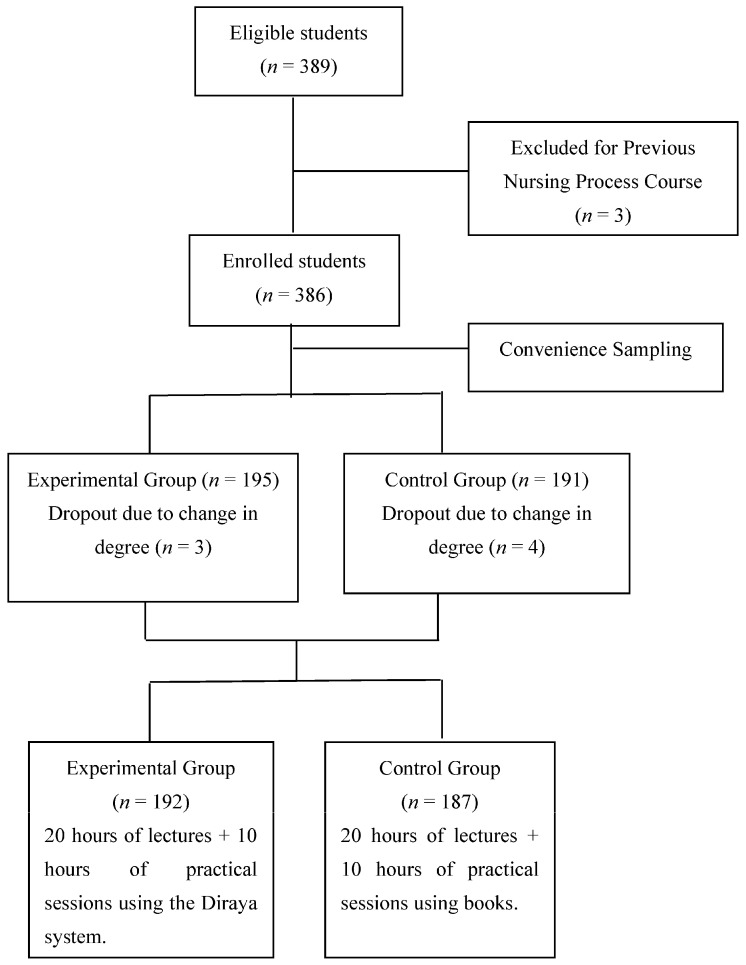
Flow of participants.

The eligible study population comprised all 379 students taking the course on “Nursing Process and Basic Care” at our Health Sciences School in one of two academic years. This course is run in the second semester of the first year of the Nursing degree course and requires 60 h of attendance, half of which are devoted to the NP. Students with previous learning in the NP were excluded from this study, which was approved by the local ethics committee (CEI-GR C-12) and followed the principles of the Declaration of Helsinki. Students were assigned to one or the other group according to the year in which they were enrolled in the course on the “Nursing Process and Basic Care”, because this subject was taught in the traditional manner (with books) in the academic year 2017–2018 and by using the Diraya AEHR system in 2018–2019. Attendance on the course automatically implied participation in the study. At the beginning of each course, the participants were informed that participation was voluntary and they could leave whenever they wished, without explanation and without an impact on their final score in this subject, and their consent was obtained. They were guaranteed that all of their personal data were confidential and would be treated in accordance with Spanish legislation on personal data protection (Law 15/13 December 1999). Participants in 2017–2018 (book learning) were informed that they would be able to use the AEHR system in one of their second-year degree courses. Figure 1 depicts the flow of participants through the study.

All participants had received a 60 h course on the use of information and communication technologies, including the corresponding computer skills, during the first semester of the first year. The students were divided into control and experimental groups for their “Nursing Process and Basic Care” course, in which 20 h of theory classes were followed by 2 h practical sessions every week for five weeks. A single teacher (L.D.-R.) delivered this course to both groups; this researcher did not participate in the analysis of results to avoid possible bias.

During the practical sessions, the control and experimental groups discussed the same clinical cases, with the same descriptions of the condition and medical history of the patients. The development of nursing care plans was based on diagnoses from the North American Nursing Diagnosis Association (NANDA), interventions from the Nursing Interventions Classification (NIC), and outcomes from the Nursing Outcomes Classification (NOC) [17,18].

#### 2.2.1. Control Group

In the control group, the five practical sessions were taught in normal classrooms using books. After reading the case reports, students assessed the patient according to the 14 Virginia Henderson Needs [19], completing complementary questionnaires, and recording vital signs. In the second session, students identified the problem and selected the diagnosis (NANDA), describing the defining characteristics and related factors. In the third session, on expected outcomes, indicators and scores were assigned (NOC) and nursing interventions and activities were selected (NIC). In the fourth week, implementation and evaluation phases were addressed and a Continuity Care Plan was prepared. Finally, a new clinical case was presented for the preparation of a complete nursing care plan, following the aforementioned phases.

#### 2.2.2. Academic Diraya Training System

In a collaborative project between our Department of Nursing and the managers of the Diraya AEHR system, this system was used to develop nine training units that included 123 fictitious patients with randomly generated first and last names. This training system was available on the Internet and administered by the teacher, who assigned each student with a code number and the names of the “virtual” patient and hospitalization unit.

#### 2.2.3. Experimental Group

The experimental group followed exactly the same curriculum and guidelines as the control group and studied the same clinical case reports. The only difference was that they used a computer with the Diraya AEHR system in the five practice sessions instead of books.

### 2.3. Sample Size Calculation

EPIDAT 3.1 software (Xunta de Galicia, Spain) was used for the sample size estimation, which was determined from the main outcome—critical thinking ability in the nursing process—using the California Critical Thinking Disposition Inventory (CCTDI), taken as a reference from a previous study [10], and we considered an α level of 0.05 and statistical power of 80%. A sample size of at least 50 participants per group was estimated.

### 2.4. Outcome Measures

All outcomes were measured before and two weeks after ending the NP practical sessions. We collected data on the acquisition of NP knowledge (conceptual competency) using a multiple-choice test with 25 questions (maximum of ten points) (Supplementary Materials): 5 questions on the characteristics of the NP method, 5 on patient assessment, 5 on nursing diagnoses, 5 on nursing care planning, and 5 on implementation and evaluation. Skill competencies were evaluated based on the final nursing care plan (maximum 10 points). The students had to identify problems and risks for the patient, prioritizing them and writing the specific label, related risk factor(s), and defining characteristics. They also had to write labels and indicators for the expected outcomes and labels for the nursing interventions, specifying individualized activities. Data were also gathered on the time required by students to finish the clinical case and on the hours of autonomous learning before the end-of-course examination. An eleven-item questionnaire with responses on a five-point Likert scale (strongly disagree = 1 to strongly agree = 5) was administered to assess attitude competencies, measuring the satisfaction of students with the program, their perception of its quality (seven items), and the difficulty experienced in applying the NP (four items) (Table 1).

### 2.5. Statistical Analysis

IBM-SPSS 22.0 (IBM Corp., New York, NY, USA) was used for the statistical analysis. Results are expressed as means with standard deviations for continuous variables and as percentages with 95% confidence intervals for categorical variables. After verifying the normality of the data distribution with the Kolmogorov–Smirnov test, a *t*-test for paired samples was used to compare knowledge and skills acquisition between control and experimental groups, and the independent-sample *t*-test to compare autonomous learning hours and students’ satisfaction with the program and their perception of its quality between groups. Univariate analysis was used to see which variables had a significant effect on the scores obtained after the program, taking into consideration conceptual, skill, and attitudinal competencies. *p* < 0.05 was considered statistically significant in all tests.

## 3. Results

**Figure 2:**
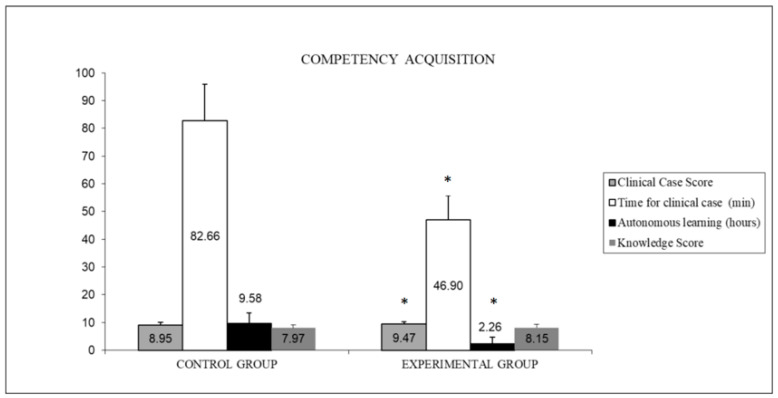
Comparison of knowledge and skill competencies between groups at the end of the program. * *p* < 0.05.

The study included 379 students, namely 302 females and 77 males, with a mean age of 20.54 ± 5.09 years. The control group contained 178 students with a mean age of 21.20 ± 5.77 years and the experimental group 192 students with a mean age of 19.91 ± 4.24 years. The groups did not significantly differ in sex distribution (*p* = 0.20), mean age (*p* = 0.01), or previous knowledge of the NP, as revealed in the multiple-choice test at baseline (*p* = 0.96). At the end of the NP course, the groups did not significantly differ in the results of a multiple-choice test on the acquisition of theoretical knowledge (*p* = 0.13); however, the experimental group achieved a significantly higher score on the clinical case (9.47 ± 0.80 vs. 8.95 ± 1.17; *p* < 0.001), took significantly less time for its completion (46.9 ± 8.76 min vs. 82.66 ± 13.14 min; *p* < 0.001), and spent fewer hours on autonomous learning to prepare for the exam (2.26 ± 2.41 vs. 9.58 ± 3.83 h; *p* < 0.001). The univariate analysis showed that the outcome basic knowledge and the interaction between basic knowledge and autonomous learning study had an effect on the final scores obtained in the program. These results are exhibited in Table 2 and Figure 2.

Satisfaction of students with the program and their perception of its quality were significantly higher in the experimental group than in the control group. Thus, as shown in Table 1, 57.6% of the experimental group vs. 32.1% of the control group strongly agreed that they were able to “learn a lot” (*p* < 0.001); 77.6% vs. 40.0%, respectively, strongly agreed that the classes were “entertaining” (*p* < 0.001); 63% vs. 37.6% strongly agreed that they were able to “apply theory to practice” (*p* < 0.001), while 0% vs. 82.6%, respectively, would have preferred to be in the other group (*p* < 0.001). There was no significant difference between the groups in their perception that the teacher was competent, that theory and practice were well connected, and that the teacher–student interaction was adequate, with the majority of all students expressing strong agreement with these affirmations.

Finally, major between-group differences were found in their perception of the difficulty in applying some phases of the NP. Specifically, they significantly differed in their responses to “It was very difficult to apply the NP” (*p* < 0.001), “The nursing diagnosis phase was very difficult” (*p* < 0.001), “The data collection assessment was very difficult” (*p* < 0.05 = 0.015), and “Planning outcomes and interventions were very difficult” (*p* < 0.001). With regard to “It was very difficult to apply the NP”, the response was “strongly agree” for 44.0% of the control group but for none of the experimental group (*p* < 0.001). In response to “The nursing diagnosis phase was very difficult”, the response was “strongly agree” for 83.50% of the control group but for none of the experimental group (*p* < 0.001 %). In response to “Planning outcomes and interventions was very difficult”, the response was “strongly agree” for 88.10% of the control group but for none of the experimental group (*p* < 0.001).

## 4. Discussion

In this study, utilization of an AEHR system for interactive computer-based classes proved to be a highly useful method for learning skills and competencies related to the NP. In comparison to students who used books alone, the students using the novel “Diraya” system scored higher on the clinical case, completed the clinical care plan faster, spent fewer hours on autonomous learning, and expressed greater satisfaction with this part of their course.

The utilization of an AEHR system has been described as the optimal approach to the acquisition of knowledge and skills [20,21,22]. Their incorporation in nursing courses has been found to enhance computer skills, increase knowledge of nursing interventions and documentation [23,24], and improve bedside nursing skills [25]. Park and Park (2015) [26] reported that a case-based computer program was an effective complementary self-study tool in an ethics course for nursing students.

Time is always at a premium in the real-life clinical setting. In comparison to the conventional approach to the NP, using books in a normal classroom, the students who used computers on our AEHR-based course required significantly less time to complete an individualized care plan. Choi, Park and Lee (2016) also observed that students taught in this way needed less time to access patient data and documentation in clinical practice, improving the productivity of nurses [23]. Besides a saving in documentation time, communication among healthcare professionals was found to be improved by this type of learning in comparison to paper-based methods [27].

In comparison to conventional learning using books, the AEHR-based method proved to be more entertaining and markedly increased skills acquisition by the students and their capacity to apply theory to practice. All phases of the NP were considered more difficult by the control group, except for the data collection phase, which showed no significant between-group difference in the perception of its difficulty.

None of the students in the experimental group would have preferred to be in the other group. Other studies found that third-year nursing students developed a positive perception of the AEHR system after using it in at least five sessions as a learning tool [22], and a constructive attitude towards this type of system is considered an important factor in its successful implementation [28].

The correct management and utilization of AEHR systems is essential to ensure care quality and patient safety [15,23,28]. In order to implement teaching methods that familiarize students with these systems and use them as a learning tool, there is a need to provide teachers with training courses in their management and to ensure adequate administrative support [29].

This study was carried out in one center during the first year of the nursing degree course and was limited to a single subject (NP). Other limitations were the lack of randomization, although there were no significant differences between the groups at baseline and the validity of the multiple-choice test to measure NP knowledge. Finally, the acquisition of competencies was only evaluated over the short term. Further research using validated outcomes is warranted on the longer-term effectiveness of this learning strategy at different stages of the degree course and for other nursing subjects. Future research controlling confounding factors should be taken into account to see the effect on the results. The major strength of this study is that it is the first to evaluate the acquisition of competencies in the NP using an AEHR system.

## 5. Conclusions

Utilization of the “Diraya” AEHR system proved to be useful to improve the acquisition by nursing students of skills, competencies, and attitudes related to the NP. Nursing faculty should become familiar with EHR systems, routinely used in the clinical setting, and they should apply these systems in teaching students to resolve clinical cases by applying the NP. This bi-directional connection between teaching and clinical practice can increase the satisfaction of students and faculty and improve the quality of care delivered to patients.

## Figures and Tables

**Table 1:** Comparison of students’ satisfaction and perception between groups.

Outcomes		CG (*n* = 187)	EG (*n* = 192)	*p*-Value
The teacher was competent.	Strongly disagree	0.00	0.00	
	Disagree	0.90	0.00	
	Neither agree nor disagree	3.70	5.50	
	Agree	27.50	16.40	0.102
	Strongly agree	67.90	78.20	
Classes were entertaining.	Strongly disagree	0.90	0.00	
	Disagree	3.70	0.00	
	Neither agree nor disagree	11.90	0.60	
	Agree	43.50	21.80	<0.001 *
	Strongly agree	40.00	77.60	
I was able to learn a lot.	Strongly disagree	3.70	0.00	
	Disagree	8.30	0.60	
	Neither agree nor disagree	0.00	4.80	
	Agree	56.00	37.00	<0.001 *
	Strongly agree	32.10	57.60	
I was able to apply the theory to the practice.	Strongly disagree	0.00	0.00	
	Disagree	3.70	0.00	
	Neither agree nor disagree	10.10	1.80	
	Agree	48.60	35.20	<0.001 *
	Strongly agree	37.60	63.00	
Theory and practice were well connected.	Strongly disagree	0.00	0.00	
	Disagree	0.00	0.00	
	Neither agree nor disagree	10.70	8.50	
	Agree	38.80	31.50	0.090
	Strongly agree	50.50	60.00	
I would like to have been in another learning group.	Strongly disagree	0.00	100.0	
	Disagree	0.00	0.00	
	Neither agree nor disagree	7.30	0.00	
	Agree	10.10	0.00	<0.001 *
	Strongly agree	82.60	0.00	
The teacher-student interaction was adequate.	Strongly disagree	0.00	0.00	
	Disagree	0.90	1.20	
	Neither agree nor disagree	3.70	7.30	
	Agree	44.70	38.80	0.861
	Strongly agree	50.70	52.70	
It was really difficult to apply Nursing Process.	Strongly disagree	1.80	0.00	
	Disagree	7.30	1.20	
	Neither agree nor disagree	21.10	84.20	
	Agree	25.70	14.50	<0.001 *
	Strongly agree	44.00	0.00	
It was really difficult the data collection assessment.	Strongly disagree	0.00	0.00	
	Disagree	0.00	0.00	
	Neither agree nor disagree	25.70	27.90	
	Agree	41.30	41.20	<0.05 *
	Strongly agree	33.00	30.90	
It was really difficult nursing diagnosis phase.	Strongly disagree	0.00	0.00	
	Disagree	0.90	0.60	
	Neither agree nor disagree	3.70	77.60	
	Agree	11.90	21.80	<0.001 *
	Strongly agree	83.50	0.00	
It was really difficult planning outcomes and interventions.	Strongly disagree	0.90	0.00	
	Disagree	0.90	0.60	
	Neither agree nor disagree	3.70	77.60	
	Agree	6.40	21.80	<0.001 *
	Strongly agree	88.10	0.00	

Independent sample *t*-test for comparison between group * *p* < 0.05; CG: control group; EG: experimental group.

**Table 2:** Comparison of knowledge and skills between groups before and after the program.

Outcomes	Control Group (*n* = 187)	Experimental Group (*n* = 192)	F	*p*-Value
Knowledge score				
Baseline	3.07 ± 1.32	3.06 ± 1.32		
Post-intervention	7.97 ± 1.13	8.15 ± 1.19	0.8	0.37
Clinical case score				
Baseline	0.00 ± 0.00	0.00 ± 0.00		
Post-intervention	8.95 ± 1.17	9.47 ± 0.80	22.2	<0.001 **
Time for clinical case (min)				
Baseline	120.00 ± 0.00	120.00 ± 0.00		
Post-intervention	82.66 ± 46.60	46.60 ± 8.76	979.96	<0.001 **
Autonomous learning (hours) *				
Pre-evaluation	9.58 ± 3.83	2.26 ± 2.41	12.09	<0.001 **

A *t*-test for paired samples was used for between-group comparisons of knowledge score, clinical score, and time for clinical cases. * Independent sample *t*-test was used to compare autonomous learning hours. ** *p* < 0.05.

## Data Availability

Not applicable.

## Supplementary Materials

The following supporting information can be downloaded at: https://www.mdpi.com/article/10.3390/jcm11102771/s1, MULTIPLE-CHOICE TEST.
