# Supplementary material for: Using “Diraya” System as a Complementary Tool in Nursing Process Education: A Controlled Clinical Study
Source: J Clin Med. 2022 May 14;11(10):2771. doi: 10.3390/jcm11102771 (PMC9143142; doi:10.3390/jcm11102771)
Supplement: Supplementary file 1 [file jcm-11-02771-s001.zip › jcm-1668807-supplementary.pdf]

Supplementary Materials

MULTIPLE-CHOICE TEST

1. **Assessment stage:**
   a. We measure the vital signs of patients.
   b. We identify the current health status of patients, their needs/problems, and possible risks they are exposed to.
   c. We gather objective data to detect the needs/problems of patients.
   d. Not necessary because physicians have already carried this out.

2. **Focused assessment:**
   a. Performed at the first contact with patients.
   b. Consists in carrying out the physical examination of a body area.
   c. Consists in focusing attention on vital signs of patients.
   d. Centers on the problems/needs detected.

3. **When is the outcome indicator score given after an intervention?**
   a. In Planning.
   b. In Assessment.
   c. In Implementation.
   d. None are correct.

4. **In the physical examination:**
   a. Palpation is the careful observation of an individual to determine physical characteristics, including size, shape, position, anatomical localization, color, movement, and symmetry
   b. Inspection is the use of touch to determine the characteristics of some body structures located beneath the skin, allowing size, shape, texture, mobility, and consistency to be determined
   c. A and B are correct
   d. None are correct

5. **Critical thinking (MARK THE INCORRECT RESPONSE):**
   a. It is controlled and deliberate thinking focused on achieving an objective.
   b. It requires data analysis and processing.
   c. It is innate and does not require practice.
   d. It is essential for application of the nursing process.

6. **In the planning stage the first step is:**
   a. To establish patient outcomes.
   b. To determine priorities.
   c. To formulate nursing diagnoses.
   d. To determine the baseline score.

7. **According to NOC taxonomy, a dependent nursing outcome is:**
   a. Status, behavior, or perception of an individual, family, or community measured over a continuum, using a measurement scale.
   b. Specific outcomes that serve to determine the situation of patients.
   c. The instrument to quantify the current status of patients and their progression over time.
   d. A and C are true.

8. **Nursing interventions and activities are performed at the stage of:**
   a. Planning
   b. Assessment
   c. Implementation
   d. Evaluation

9. **From the following, which is a subjective datum?**
   a. The patient drinks three glasses of water/day.

    b. The caregiver of the patient says that he/she defecates twice a day.
    c. The patient says he/she has difficulty breathing.
    d. The patient takes a siesta of 1 h/day.

10. **The objective of the nursing care process is:**
    a. Treatment of the disease.
    b. Response of individuals, family, or community to health problems.
    c. Correct functioning of organs and systems.
    d. Application of techniques to patients.

11. **In order to perform interventions, nursing professionals:**
    a. Carry out an assessment, monitor orders and equipment, wash hands, use gloves if required, identify patients.
    b. Always explain what they are going to do. Provide safety and privacy.
    c. Wash hands, use gloves if required, identify patients.
    d. A and B are true.

12. **The care continuity report:**
    a. Is given at admission.
    b. Provides information on the medication patients must take.
    c. Serves to inform patients/relatives on the care that must continue at home.
    d. All are true.

13. **The source of data for nursing assessment are:**
    a. Medical records are the most important because they gather a large amount of information.
    b. Individuals, their environment (relatives, caregivers), and professional records.
    c. Mainly individuals, who can always give the most information.
    d. Individuals and their family environment.

14. **Nursing interventions:**
    a. Can be independent, dependent, direct, or indirect.
    b. Can be independent, dependent, direct, indirect, focused, and general.
    c. Can be independent or dependent.
    d. Can be technical, focused, direct, or independent.

15. **Problems of collaboration:**
    a. Nursing professionals do nothing because it is not their responsibility.
    b. Nursing professionals monitor complications derived from a medical diagnosis or treatment.
    c. Nursing professionals administer prescribed treatments.
    d. B and C are correct.

16. **After performing an intervention:**
    a. There is no need to do anything because it is already described in the planning.
    b. We report it at the change of the shift.
    c. We record it, including the timeline and responses of patients.
    d. We record it before it is performed to avoid oversights.

17. **Real nursing diagnoses:**
    a. Describe human responses to health problems/life processes that may develop in a person, family, or community.
    b. Describe human responses to health problems in a person, family, or community.
    c. Describe human responses to increase the wellbeing and health potential of a person, family, or community.
    d. Describe human responses to health problems/life processes in a person, family, or community.

18. **In the NANDA-I taxonomy, a nursing diagnosis of risk consists of:**
    a. Name or label, definition, defining characteristics, and risk factors.
    b. Name or label, code, definition, risk factors, and defining characteristics.

c. Name or label, code, definition, and risk factors.
d. Name or label, code, definition, defining characteristics, and related factors.

19. With respect to standardized care plans (MARK THE INCORRECT RESPONSE):
a. They are designed for a group of patients with common needs.
b. They include the nursing diagnoses associated with a specific medical disorder; therefore, further diagnoses cannot be added.
c. They ensure the fulfillment of minimal nursing care requirements.
d. They optimize the time of the professional.

20. How is a nursing diagnosis correctly formulated according to the PES format?
a. LABEL expressed by RELATED FACTORS associated with DEFINING CHARACTERISTICS
b. LABEL associated with RELATED FACTORS expressed by DEFINING CHARACTERISTICS.
c. LABEL expressed by DEFINING CHARACTERISTICS associated with RELATED FACTORS.
d. LABEL expressed by RISK FACTORS associated with RELATED FACTORS.

21. The nursing process:
a. Consists of five inter-related stages: assessment, diagnosis, planning, implementation, and evaluation.
b. Consists of five completely independent stages: assessment, diagnosis, planning, implementation, and evaluation.
c. Consists of four stages related to each other: assessment, diagnosis, planning, and evaluation.
d. Consists of five stages related to each other: assessment, data analysis, diagnosis, planning, and evaluation.

22. Gathering, validating, classifying, and recording data are stages of:
a. Planning
b. Assessment
c. A and B are correct
d. Identification

23. Did the patient achieve what was planned? What stage of the nursing process does it correspond to?
a. Assessment
b. Evaluation
c. Implementation
d. Diagnosis

24. In nursing intervention 7310 *Patient care at admission*, the following activities were performed, among others (MARK THE INCORRECT RESPONSE):
a. Introducing yourself and your care function.
b. Obtaining the orders of physicians on the care to be delivered to patients.
c. Performing the nursing assessment.
d. Preparing the care continuity report.

25. Can a real nursing diagnosis become a risk diagnosis?
a. No, a real diagnosis can only be maintained or resolved.
b. Yes, when it is resolved but risk factors persist.
c. Yes, when it is resolved but defining characteristics persist.
d. No, it can only become a health promotion diagnosis.
